# Supplementary material for: Are Perceptions of Government Intervention Related to Support for Prevention? An Australian Survey Study
Source: Healthcare (Basel). 2023 Apr 27;11(9):1246. doi: 10.3390/healthcare11091246 (PMC10177783; doi:10.3390/healthcare11091246)
Supplement: Supplementary file 1 [file healthcare-11-01246-s001.zip › healthcare-2226284-supplementary.pdf]

Australian Perceptions of Prevention Survey (AUSPOPS)
Questionnaire – Final
25 September 2018

CALL OUTCOMES AND RR1

**USE STANDARD BUT SHOW NO-ONE 18 PLUS IN HOUSEHOLD

**USE STANDARD RR1 AND RR2 BUT ADD OBJECTED TO BEING CALLED ON A MOBILE PHONE TO RR1

PARTICIPANT INFORMATION SAMPLE FIELDS

**USE STANDARD

INTRODUCTION

*(TIMESTAMP1)

*(ALL)
INTRO Good morning/afternoon/evening. My name is (....) and I'm calling from the Social Research Centre on behalf of the University of Sydney. The University is doing a study on how we value health as a community and as individuals. The study asks about your views on how the government spends public money and makes policy relating to the community's health.

IF NECESSARY: The survey is mainly about your views on how the government spends public money and makes policy relating to the community's health. The results from this survey will be used to support and guide policies and programs that aim to improve the lives of all Australians.

IF NECESSARY: This survey is not associated with any political party.

*(LANDLINE SAMPLE) (SAMTYP=1)
S1 To help with this important study we'd like to arrange a short interview with the person aged 18 or over who is going to have the next birthday. May I speak to that person please?

IF NECESSARY: Good morning/afternoon/evening. My name is (....) and I'm calling from the Social Research Centre on behalf of the University of Sydney. The University is doing a study on how we value health as a community and as individuals.

1. Continue
2. Household refusal (ATTEMPT CONVERSION / RECORD REASON) (GO TO RR1)
3. Respondent refusal (GO TO RR1)
4. Queried about how telephone number was obtained (GO TO ATELQ)
5. Needs more information (GO TO AINFO)

*(MOBILE PHONE SAMPLE) (SAMTYP=2)
S5 For this survey we are interested in talking to people aged 18 or over. Can I check, are you aged 18 years or over?

1. Yes
2. No (GO TO TERM1)
3. Refusal (GO TO RR1)

*(MOBILE PHONE SAMPLE) (SAMTYP=2)
S3 Could I also just check whether it is safe for you to take this call at the moment … If not, we'd be happy to call you back when it is more convenient for you.

1. Safe to take call
2. Not safe to take call
3. Refusal (GO TO RR1)

*(NOT SAFE TO TAKE CALL) (S3=2)

S4 Do you want me to call you back on this number or would you prefer I call back on your home phone?

1. This number (MAKE APPOINTMENT)
2. Home phone (MAKE APPOINTMENT, RECORD HOME PHONE NUMBER)
3. Respondent refusal (GO TO RR1)

*(MOBILE PHONE SAMPLE AGED 18 OR OVER) (SAMTYP=2 AND S5 = 1)

S6 Can you please tell me which state or territory you‘re in?

1. NSW
2. VIC
3. QLD
4. SA
5. WA
6. TAS
7. NT
8. ACT
9. (Refused) (GO TO TERM2)

*(ALL)

S7 This study is mainly about your opinions. There are no right or wrong answers. If I come to any question you prefer not to answer, just let me know and I'll skip over it. You can withdraw from the study at any point and the information collected will not be retained, or you may complete the rest of the interview at another time. All interviews are voluntary, and we will treat all information you give in strict confidence.

This interview should take around 15-20 minutes. I'll try and make it as quick as I can.

Are you happy to continue?

1. Continue
2. Respondent refusal (ATTEMPT CONVERSION / RECORD REASON) (GO TO RR1)
3. Queried about how telephone number was obtained (GO TO ATELQ)
4. Needs more information (GO TO AINFO)

*(ALL)

MONREC This call may be monitored or recorded for quality assurance purposes. Is that ok?

1. Yes
2. No

*(QUERIED HOW TELEPHONE NUMBER WAS OBTAINED)

ATELQ Your phone number has been randomly generated by computer. We find that this is the best way to obtain a representative sample and to make sure we get opinions from a wide range of people.

1. Snap back to previous question (Intro / S1 / S7)

*(WANTS MORE INFORMATION)

AINFO IF NECESSARY: The survey is mainly about your views on how the government spends public money and makes policy relating to the community's health. The results from this survey will be used to support and guide policies and programs that aim to improve the lives of all Australians.

IF NECESSARY: This survey is not associated with any political party.

1. Snap back to previous question (Intro / S1 / S7)

SECTION D: BARRIERS TO PREVENTION

*(ALL)

D1 As far as you are aware, how much of an effect do the following things have on people's health? Please use a scale from 1 to 5, where 1 means 'no effect at all' and 5 is 'a very large effect'.

(ROTATE)
(STATEMENTS)

a) The type of food a person eats
b) The amount of physical activity a person does
c) A person's genetic make-up
d) A person's financial circumstances
e) Whether or not a person smokes cigarettes
f) Whether or not a person drinks alcohol
g) Where in Australia someone lives
h) Access to health and hospital services
i) Access to bike paths
j) Having activities to promote health in the workplace
k) Being able to afford to go to a gym to exercise

(RESPONSE FRAME)

1. No effect at all
2. A small effect
3. A moderate effect
4. A large effect
5. A very large effect
6. (Don't know)
7. (Refused)

*(TIMESTAMP3)

SECTION C: VALUE OF PREVENTION

(ROTATE C3A, C3B, C3C, C3D & C3E) (SHOWN ONLY FOUR)

*(ALL)

C3a **PROGRAMMER NOTE: Show following text for first question

Which one of the following two health initiatives do you think would make the most difference to improving the community's health?

1. Subsidising drugs that lower blood pressure, **OR**
2. Setting limits of salt in processed food to lower blood pressure
3. (Don't know)
4. (Refused)

*(ALL)
C3b *PROGRAMMER NOTE: Only show following text after first question has been asked

And how about…

IF NECESSARY: Which one of the following two health initiatives do you think would make the most difference to improving the community's health?

1. Providing low cost gym membership, **OR**
2. Building a network of walking and cycle paths
3. (Don't know)
4. (Refused)

*(ALL)
C3c PROGRAMMER NOTE: Only show following text after first question has been asked

And how about…

IF NECESSARY: Which one of the following two health initiatives do you think would make the most difference to improving the community's health?

1. Taxing processed food with high sugar or fat content, **OR**
2. Subsidising operations for people who are obese
3. (Don't know)
4. (Refused)

*(ALL)
C3d PROGRAMMER NOTE: Only show following text after first question has been asked

And how about…

IF NECESSARY: Which one of the following two health initiatives do you think would make the most difference to improving the community's health?

1. Funding alcohol treatment centres, **OR**
2. Placing restrictions on alcohol advertising
3. (Don't know)
4. (Refused)

*(ALL)
C3e PROGRAMMER NOTE: Only show following text after first question has been asked

And how about…

IF NECESSARY: Which one of the following two health initiatives do you think would make the most difference to improving the community's health?

1. Increase access to fruit and vegetables, **OR**
2. Subsidise medications to lower cholesterol
3. (Don't know)
4. (Refused)

*(TIMESTAMP4)

SECTION E: RESPONSIBILITIES FOR PREVENTION

*(ALL)

E1 To what extent do you think each of the following have a role in maintaining people's health? Please use a scale from 1 to 5, where 1 means 'no role at all' and 5 is 'a very large role'.

(ROTATE)
(STATEMENTS)

b) Government
c) Parents
d) People themselves
e) GPs, nurses, pharmacists
f) Employers
g) Food manufacturers
h) Schools
i) Private health insurers
j) Alcohol manufacturers

Would you say…?

(RESPONSE FRAME)

1. No role at all
2. A small role
3. A moderate role
4. A large role
5. A very large role
6. (Don't know)
7. (Refused)

*(ALL)

E2 For each of the following government initiatives, please tell me whether you think it shows the government going too far, not far enough or having about the right amount of involvement in helping people be healthy?

IF NECESSARY: Some of these initiatives **have** been introduced by the government, whilst others **could** be introduced to help people be healthy and prevent disease.

(ROTATE)
(STATEMENTS)

a) Plain packaging for tobacco products
b) Bans on smoking in cars with children
c) Lower speed limits (30km/hr) in high pedestrian areas
d) Restrictions on advertising unhealthy foods to children
e) Restrictions on alcohol advertising
f) Taxing soft drink
h) Setting salt limits on processed food
j) Compulsory immunisation at school entry
k) Laws setting limits on working hours
l) Creation of bike lanes separated from cars
m) Removing advertising for unhealthy food and drinks in places owned by the Government (such as train stations)
n) Restrictions on sports sponsorship by companies that sell unhealthy food and drinks
p) Banning venues with an alcohol license from selling cigarettes

(RESPONSE FRAME)

1. Too far
2. About the right amount
3. Not far enough
4. (Don't know)
5. (Refused)

*(TIMESTAMP6)

*(ALL)
E3 In general, do you think Australia has too much, too little or about the right amount of government regulation and policies in place to help people be healthy?

IF NECESSARY: By regulation we mean things like bans, taxes and restrictions

1. Too much
2. About the right amount
3. Not enough
4. (Don't know)
5. (Refused)

*(ALL)
E5 People in our society often disagree about how far to let individuals go in making decisions for themselves. Do you agree or disagree with the following statements?

(ROTATE)
(STATEMENTS)

a) Sometimes government needs to make laws that keep people from harming themselves
b) The government interferes far too much in our everyday lives
c) It's not the government's business to try to protect people from themselves
d) Government should put limits on the choices individuals can make so they don't get in the way of what's good for society
e) Maintaining the community's health requires a combination of both government regulation and personal responsibility.
f) Limiting the advertising and sale of unhealthy products make it easier for people to make healthy choices.
g) It is not worth spending money on prevention because people will do what they want anyway
h) Government regulation on health has made Australia a nanny state.

*PROGRAMMER NOTE: ONLY SHOW THE FOLLOWING INTERVIEWER NOTE FOR STATEMENT H.

IF NECESSARY: The term 'nanny state' means that a government or its policies are overprotective and interfere with personal choice

PROBE: Is that agree / disagree or strongly agree / disagree?

(RESPONSE FRAME)

1. Strongly disagree
2. Disagree
3. Neither agree nor disagree
4 Agree
5. Strongly agree
6. (Don't know)
7. (Refused)

*(ALL)
E6 Sometimes the government puts a tax on certain products that can negatively affect people's health to regulate their use. In general, do you support or oppose the idea of the government putting a tax on a product that can negatively affect people's health?

PROBE: Is that support / oppose or strongly support / oppose?

1. Strongly oppose
2. Oppose
3. (Neither support nor oppose)
4. Support
5. Strongly support
6. (Don't know)
7. (Refused)

SECTION H: PERSONAL HEALTH

*(ALL)
H1 The next questions are about your own health.

Would you say your health is… (READ OUT)

1. Excellent
2. Very good
3. Good
4. Fair
5. Poor
6. (Don't know)
7. (Refused)

*(ALL)
H3 In the past week, on how many days have you done a total of 30 minutes or more of physical activity which was enough to raise your breathing rate?

This includes sport, exercise, brisk walking, cycling for recreation or transport, BUT NOT including housework or physical activity as part of your job.

INTERVIEWER NOTE: Repeat "How many days in the past week…' as necessary

1. Days given (SPECIFY) (RANGE 0 to 7)
2. (Don't know)
3. (Refused)

*(ALL)
H4 Do you currently smoke cigarettes on a daily or weekly basis?

IF NECESSARY: By cigarettes we mean factory-made or roll-your-own cigarettes

1. Yes
2. No
3. (Don't know)
4. (Refused)

*(ALL)
H6 How often did you have a drink containing alcohol in the past year? (READ OUT)

1. Every day
2. 3-6 days a week
3. 1-2 days a week
4. 2-3 days a month
5. Once a month
6. Less than once a month
7. Never
8. (Don't know)
9. (Refused)

*(ALL)
H10 Have you been told by a doctor or nurse that you currently have any of the following long-term health conditions…..(READ OUT)

(MULTIPLE RESPONSE)
(ROTATE)

1. Arthritis
2. Asthma
3. Heart disease
4. Stroke, or at risk of a stroke
5. Chronic kidney disease
6. Cancer of any kind
7. Depression
8. Type 2 Diabetes
9. Oral Disease (e.g. Gum disease)
10. Osteoporosis
11. (None) ^s
12. (Don't know) ^s
13. (Refused) ^s

*(TIMESTAMP8)

DEMOGRAPHICS AND WEIGHTING

*(ALL)
DEM1 We're nearly finished now. Just a final few questions to make sure we've spoken to a good range of people...

Including yourself, how many people aged 18 years and over live in your household?

1. Number given (SPECIFY) (RANGE 1 to 20) *(DISPLAY "UNLIKELY RESPONSE" IF > 10)
2. (Don't know)
3. (Refused)

*(ALL)
DEM2 Would you mind telling me how old you are?

1. Age given (SPECIFY) (Allowable range: 18 TO 120)
2. (Refused)

*(REFUSED AGE)
DEM3 No problem, would you mind telling me which of the following age groups you are in? (READ OUT)

1. 18 - 24 years
2. 25 - 34 years
3. 35 - 44 years
4. 45 - 54 years
5. 55 - 64 years
6. 65 - 74 years
7. 75+ years
8. (Refused)

*(ALL)
DEM4 RECORD GENDER

1. Male
2. Female

*(ALL)
DEM5 In which country were you born?

1. Australia
2. Canada
3. China (excluding Taiwan)
4. Croatia
5. Egypt
6. Fiji
7. Germany
8. Greece
9. Hong Kong
10. Hungary
11. India
12. Indonesia
13. Ireland
14. Italy
15. Lebanon
16. Macedonia
17. Malaysia
18. Malta
19. Netherlands (Holland)
20. New Zealand
21. Philippines
22. Poland
23. Serbia / Montenegro
24. Singapore
25. South Africa
26. Sri Lanka
27. Sudan
28. United Kingdom (England, Scotland, Wales, Nth Ireland)
29. USA
30. Vietnam
31. Other (SPECIFY)
32. (Refused)

*(ALL)
DEM6 Do you usually speak a language other than English at home?

1. Yes
2. No
3. (Don’t know)
4. (Refused)

*(ALL)
DEM7 Are you from an Aboriginal and/or Torres Strait Islander background?

1. Yes
2. No
3. (Don’t know)
4. (Refused)

*(ALL)
DEM8 Which one of the following BEST describes your employment situation? (READ OUT)

1. Employed (FT, PT, Self-employed, casual)
2. Unemployed
3. Retired/pension
4. Student
5. Home duties
6. Other (SPECIFY)
7. (Don’t know)
8. (Refused)

*(EMPLOYED, DEM8=1)
DEM9 And, what is your current occupation?

PROBE: Main duties and job title

1. Managers
2. Professionals
3. Technicians and trades workers
4. Community and personal service workers
5. Clerical and administrative workers
6. Sales workers
7. Machinery operators and drivers
8. Labourers
9. Other (SPECIFY)
10. (Don’t know)
11. (Refused)

*(ALL)
DEM10 What is the highest level of education you have completed? PROMPT IF REQUIRED

INTERVIEWER NOTE: If Year 12 or less, probe for trade qualifications / TAFE certificates

1. Primary school
2. Year 7-9
3. Year 10
4. Year 11
5. Year 12

6. Trade/apprenticeship
7. Other TAFE/ Technical certificate
8. Diploma
9. Bachelor degree
10. Post-graduate degree
11. Other (SPECIFY)
12. (Don't know)
13. (Refused)

*(ALL)
DEM11 Are you currently receiving income support or a pension from the government (e.g. aged, disability, income support)?

1. Yes
2. No
3. (Don't know)
4. (Refused)

*(ALL)
DEM12 Do you have private health insurance?

1. Yes
2. No
3. (Don't know)
4. (Refused)

*(MOBILE SAMPLE) (SAMTYP=2)
W1 Now just a question or two about your use of telephone services.

Is there at least one working fixed line telephone inside your home that is used for making and receiving calls?

1. Yes
2. No
3. (Don't know)
4. (Refused)

*(LANDLINE SAMPLE, MOBILE SAMPLE WITH LANDLINE) (SAMTYP=1 OR ((SAMTYP=2 AND W1 = 1))
W2 How many residential phone numbers do you have in your household, not including lines dedicated to faxes, modems or business phone numbers? Do not include mobile phones.

INTERVIEWER NOTE: If needed explain as how many individual landline numbers are there at your house that you can use to make and receive calls?

1. Number of lines given (SPECIFY) RECORD WHOLE NUMBER (ALLOWABLE RANGE 1 TO 15) *(DISPLAY "UNLIKELY RESPONSE" IF >3)
2. (Don't know)
3. (Refused)

*(LANDLINE SAMPLE) (SAMTYP=1)
W3 Do you also have a working mobile phone?

1. Yes
2. No
3. (Don't know)
4. (Refused)

*(ALL)
DEM13 And finally, can I also have your postcode please?

IF NECESSARY: It is important that we collect this information so we can analyse results at a local level

(DISPLAY SAMPLE POSTCODE)

1. Sample postcode correct *SAMTYPE=1 ONLY
2. Correct sample postcode (SPECIFY) (Allowable range: 800 TO 9729) *SAMTYPE=1 ONLY
3. Enter postcode (SPECIFY) (Allowable range: 800 TO 9729) *SAMTYPE=2 ONLY
4. (Don't know)
5. (Refused)

*(TIMESTAMP9)

CLOSE

*(ALL)
END1 That's the end of the survey. Thanks for your time. This survey is carried out in compliance with the Privacy Act, and the information you have provided will only be used for research purposes. Our Privacy Policy is available via our website (www.srcentre.com.au).

Just in case you missed it, my name is (…) and this survey was conducted by the Social Research Centre.

CLOSE SUITABLY

TERMINATION SCRIPTS

TERM1 Thanks anyway, but for this study we need to speak to people aged 18 or over. Thanks for being prepared to help out.

TERM2 That's okay, but to take part in this study I need to confirm which state / territory you are in.

ALLTERM

	**Detailed outcome**	**Summary outcome**	**AAPOR code**	**AAPOR category**
	Complete	Complete	1.0	Complete
S1=2	Household refusal	Refusal	2.111	HH refusal
S1=3	Respondent refusal	Refusal	2.112	Known respondent refusal
S5=2	Mobile – not over 18	Out of scope	4.7	No eligible respondent
S5=2	Mobile – refused age screener	Refusal	3.21	Screener not complete
S3=3	Mobile – refused safety question	Refusal	3.21	Screening not complete

S4=3	Respondent refusal	Refusal	2.112	Known respondent refusal
S6=9	Refused state	Refusal	3.21	Screening not complete
S7=2	Respondent refusal	Refusal	2.112	Known respondent refusal

*(TIMESTAMP10)

Table S1: Significance for bivariate analyses of all outcome and exposure variables across demographic variables

Characteristic	**Nanny scale**	**E5a**	**E5d**	**E5e**	**E5f**	**Tobacco scale**	**Diet scale**	**Intervention preference**
**Gender**	<0.001	0.055	0.352	0.108	0.086	0.146	<0.001	0.663
**Age (<35yrs)**	<0.001	0.650	0.992	0.022	0.998	<0.001	<0.001	0.142
35-<55yrs	0.757	0.460	0.924	0.505	0.980	0.864	<0.001	0.690
55+yrs	<0.001	0.369	1.00	0.084	0.957	<0.001	<0.001	0.079
**Metro vs Regional**	<0.001	0.089	0.420	0.033	0.097	0.371	0.731	0.796
**Country of birth**	0.060	0.082	0.056	0.312	0.012	0.002	0.251	0.045
**Education (High School)**	<0.001	<0.001	0.007	<0.001	0.169	0.444	0.049	0.001
Post-secondary	0.208	0.166	0.437	0.001	0.955	0.231	0.502	0.173
Uni Degree	<0.001	<0.001	0.024	<0.001	0.106	0.774	0.018	<0.001
**Low disadvantage vs high disadvantage**	<0.001	0.116	0.200	0.092	<0.001	0.107	0.074	0.216
